# Antibacterial Effects of Curcumin Nanocrystals against *Porphyromonas gingivalis* Isolated from Patients with Implant Failure

**DOI:** 10.3390/clinpract12050085

**Published:** 2022-10-06

**Authors:** Solmaz Maleki Dizaj, Hojjat Shokrgozar, Javad Yazdani, Mohammad Yousef Memar, Simin Sharifi, Mohammad Ali Ghavimi

**Affiliations:** 1 Dental and Periodontal Research Center, Tabriz University of Medical Sciences, Tabriz 5166, Iran; 2 Department of Oral and Maxillofacial Surgery, Faculty of Dentistry, Tabriz University of Medical Sciences, Tabriz 5166, Iran; 3 Infectious and Tropical Diseases Research Center, Tabriz University of Medical Sciences, Tabriz 5166, Iran

**Keywords:** antibacterial effects, curcumin, nanocrystals, *Porphyromonas gingivalis*, implant failure

## Abstract

Background. Despite their benefits, dental implants may sometimes fail for a diversity of causes; the most common reasons of failure are infection and bone loss. *Porphyromonas gingivalis* (*P.* *gingivalis*) bacteria show a major role in peri-implantitis infection and dental implant failure. Methods. In this study, the prevalence of *P. gingivalis* isolated from the gingival crevicular fluid (GCF) of fifteen Iranian patients with implant failure (more than 1/3 of the implant length), who had average oral and dental hygiene and no antibiotic use for at least one month, was determined. Moreover, the antimicrobial effects of curcumin nanocrystals against isolated *P.* *gingivalis* were investigated. The collected samples from patients were transferred to a microbiology laboratory to culture. The presence of *P. gingivalis* in the culture media was confirmed using a trypsin reagent test. An isolate from a patient with the highest colony count was selected to evaluate the antibacterial effects of curcumin nanoparticles. The inhibition zone diameter, minimum inhibitory concentration (MIC), and minimum bactericidal concentration (MBC) were determined. Results. Out of fifteen patients, eight (53.33%) were positive for the presence of *P.* *gingivalis*. The results of the microbial tests showed that curcumin nanoparticles had an MIC of 6.25 µg/mL and an MBC of 12.5 µg/mL. Conclusions. The use of curcumin nanoparticles may control the bacterial infection around the implant.

## 1. Introduction

After an implant is implanted into the jaw, it needs a healing time before the placement of a crown. Throughout this time, the implant fuses with the surrounding bone. This procedure is known as osseointegration [1,2].

Dental implants, despite their many advantages, may sometimes fail for a variety of reasons [3]. The implant has been shown to have consistently higher levels of inflammation than the natural tooth, which is why approving solutions capable of limiting this effect and keeping the health of peri-implant tissues for a long time is essential [4]. Peri-implantitis is an infectious disease that occurs around the implant and inside the gums. It is generally the outcome of poor dental hygiene following a dental implant procedure leading to more bone loss and implant failure [3,5]. In patients with low mandibular bone density or dental damage after implant surgery, implant failure may occur due to the lack of integration of the implant and jawbone [4]. The mechanical properties of the implant may also play a major role in implant failure [3,5]. Poor implant planning including implant design and diameter and occlusal overloading are the common variables related to implant fracture [5,6,7]. The wrong positioning, wrong prosthodontics plan, and too short biologic width also are other reasons for implant failure [3].

Recently, modern techniques of stem cell stimulation and bone neo-apposition with grafts are progressing; they also offer good opportunities in implant re-osseointegration. [8] To improve the stability and osseointegrations of implants, the dentistry industry uses a variety of scaffolds and growth factors [8,9]. Bioactive-molecule-loaded scaffolds show a main role in the promotion of stability and osseointegration in implants [8].

As mentioned, infection is known as one of the critical reasons in this regard [10,11]. *Porphyromonas gingivalis* (*P. gingivalis*) is recognized as one of the leading causes of dental implant failure [12]. Antibiotics are commonly used to control the infection of *P. gingivalis* [13]. The ineffectiveness of antibiotics against *P. gingivalis* is due to the alterations in the phenotype of the bacterium after its entry into the cells [13,14]. Clinical trials on periodontitis patients treated with antibiotics showed that the antibiotic therapy alone could not completely eliminate *P. gingivalis* [13]. Numerous side effects of synthetic antibiotics as well as the spread of drug resistance also lead to a tendency to use natural antimicrobials [11,15,16].

Curcumin is the active ingredient of turmeric plants and has powerful antimicrobial and anti-inflammatory properties. It is recognized for its medicinal properties such as anti-inflammatory, antioxidant, antimicrobial, hepatoprotective, immunostimulant, antiseptic, and antimutagenic [17]. Owing to these properties, it is a useful material in dentistry as well. It shows effectiveness in the treatment of periodontal diseases and oral cancers. It can also be applied as an active part in local drug delivery systems in gel form for dental uses. Curcumin or its nanoformulations can be used both in local and systemic forms [17,18].

A recent study showed that curcumin is very sensitive to *P. gingivalis* with the minimum inhibitory concentration (MIC) of 12 µg/mL [19]. Despite its unique properties, curcumin has minimal solubility in water. According to reports, nanocrystallization of curcumin or its loading into nanoparticles increases its solubility and antimicrobial potency [20,21].

In this study, we investigated the effects of curcumin nanocrystals on microbial contamination of *P. gingivalis* isolated from the gingival crevicular fluid (GCF) of Iranian patients with implant failure.

## 2. Materials and Methods

### 2.1. Ethical Considerations

The ethics code was received from the Ethics Committee of Tabriz University of Medical Sciences, and all steps were carried out after obtaining the code (IR.TBZMED.REC.1399.194). All participants filled out the written informed consent.

### 2.2. Materials

Curcumin powder was obtained from Sigma-Aldrich (Oakville, ON, Canada), and ethanol and hexane were purchased from Merck Company (Darmstadt, Germany). Thioglycolate broth, Brucella agar, defibrinated sheep blood, horse serum, vitamin K1, calcitonin antibiotic, trypsin reagent, amoxicillin, metronidazole, ciprofloxacin, amikacin, and gentamicin were purchased from Gibco, Ireland.

### 2.3. Methods

#### 2.3.1. Preparation and Characterization of Curcumin Nanocrystals

Curcumin powder (80 mg) was dissolved in 10 mL of ethanol (Merck, Germany/Deutschland). Then, hexane (300 mL) was quickly added to the curcumin solution. A yellow suspension was obtained. The solvents were evaporated using a rotary evaporator (IKA^®^-Werke GmbH & Co- Germany/Deutschland). Finally, a spray drier (Shanghai, China/Shanghai) was used for spray-drying of curcumin nanocrystals by the following operation: an outlet temperature of 80 °C, inlet temperature of 150 °C, and liquid feed rate of 1.5 mL/min. 

##### Characterization of Particle Size

A dynamic light scattering (DLS) device was used (Malvern, Worcestershire, England) for measuring the mean particle size of curcumin nanoparticles. The following operation was used for the DLS device: an argon laser beam at 633 nm and a 90° scattering angle at 25 °C.

##### Sample Size

The sample size was determined according to the results of Mombelli et al. [22] and considering the first type error (equal to 0.05) and the power of 80%. Then, a sample number equal to 15 was obtained.

#### 2.3.2. Inclusion Criteria

The patients referred to the educational and therapeutic center of Tabriz University of Medical Sciences Dental School due to implant failure (more than 1/3 of the implant length), who had average oral and dental hygiene and had not used antibiotics for at least one month. The oral and dental hygiene status was determined using the simplified oral hygiene index (SOHI) based on the debris index (DIS) and the calculus index (CIS) and considering the participant’s age [23].

#### 2.3.3. Exclusion Criteria

Patients with a history of smoking or drug addiction, those with a history of certain diseases or a history of taking certain drugs, and those whose oral and dental hygiene was very poor were excluded from the study.

#### 2.3.4. Sampling of *P. gingivalis*

In this study, 15 patients with dental implant failure were selected according to the inclusion and exclusion criteria of the patients referred to the Department of Oral and Maxillofacial Surgery, Faculty of Dentistry, Tabriz University of Medical Sciences, Tabriz, Iran. First, the surface of the failed implant was cleaned with sterile gauze to remove salivary secretions and any other contamination. The patient’s gingival crevice fluid (from the failed implant) was then sampled using a sterile filter paper and placed in a thioglycollate broth culture medium. The samples were transferred to the microbiology laboratory in less than 30 min.

#### 2.3.5. Microbiological Tests

The samples were vortexed for 30 s to separate the bacteria. A Brucella agar medium containing 5% defibrinated sheep blood, hemin, vitamin K1, bacitracin, colistin sulfate, and nalidixic acid was used for the isolation of bacteria. The plates were incubated for three days at 37 °C under anaerobic conditions. Then, the presence of *P. gingivalis* in the culture media was confirmed using a trypsin reagent test, which is a diagnostic test for *P. gingivalis* [24].

#### 2.3.6. Sensitivity of Bacteria to Nanoparticles

The disk diffusion method was used to determine the sensitivity of *P. gingivalis* to the curcumin nanoparticles. First, a 0.5-McFarland suspension was prepared from the bacterial isolate, and then a uniform lawn culture was performed on the surface of Brucella agar supplemented with hemin (5 µg/mL), vitamin K_1_ (1 µg/mL), and laked sheep blood (5%). In the next step, sterile blank disks were immersed in the curcumin nanoparticle’s suspension (concentrations of 50, 25, 12.5, 6.25, and 3.12 µg/mL). The disks were placed on the agar surface. Antibiotic disks of metronidazole (5 µg/mL) were used as a positive control, and a blank disk immersed in water was used as a negative control. The plates were incubated for 42 h at 37 °C, and then the growth inhibition zones around the disks were measured.

#### 2.3.7. Determination of MIC

To determine the MICs of the nanoparticles against *P. gingivalis*, broth microdilution method was performed using Brucella broth supplemented with hemin (5µg/mL), vitamin K1 (1 µg/mL), and lysed horse blood (5%) in the presence of a serial concentration of nanoparticles (50, 25, 12.5, and 6.25 µg/mL concentrations). Metronidazole antibiotic was used as a control (positive control). Wells containing water were considered as a negative control. The wells were incubated for 48 h at 35 °C and then examined for microbial growth turbidity. Visual turbidity detection was used to determine the MIC.

#### 2.3.8. Determination of MBC

Ten microliters of the three pre-MIC wells was separately cultured on Brucella agar supplemented with hemin (5 µg/mL), vitamin K1 (1 µg/mL), and lysed sheep blood (5%). After 48 h, the lowest concentration of suspension in which the bacterium did not grow was reported as the MBC.

### 2.4. Statistical Data Analysis Method

The results were reported using descriptive statistics. In order to compare the antibacterial properties, one-way ANOVA was used. For data analysis, SPSS software version 2020 was used. A probability value of less than 0.05 was considered a significant level.

## 3. Results

### 3.1. Mean Particle Size of Curcumin Nanoparticles

**Figure 1:**
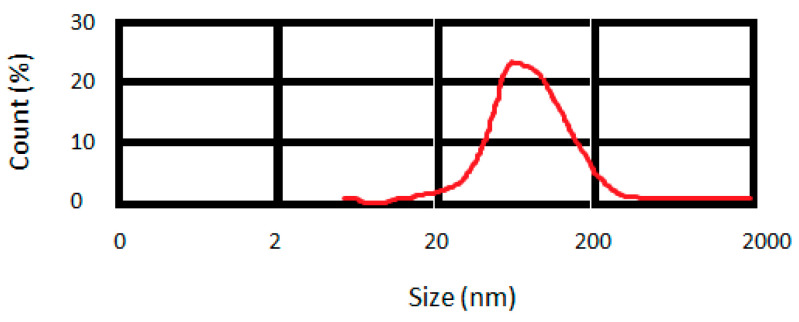
Distribution of particle size for prepared curcumin nanoparticles.

The prepared curcumin nanoparticles exhibited a mean particle size of 95 nm (Figure 1).

### 3.2. Prevalence of P. gingivalis in Selected Patients

Among the fifteen patients (eight men and seven women), eight patients (53.33%) were positive for the prevalence of *P. gingivalis*. Among the positive patients, five were men (62%) and three were women (38%). Two patients (25%) were in the age group of 20–35, two in the age group of 35–50, and four (50%) in the age group of 50–65 years old.

### 3.3. Sensitivity of P. gingivalis

**Figure 2:**
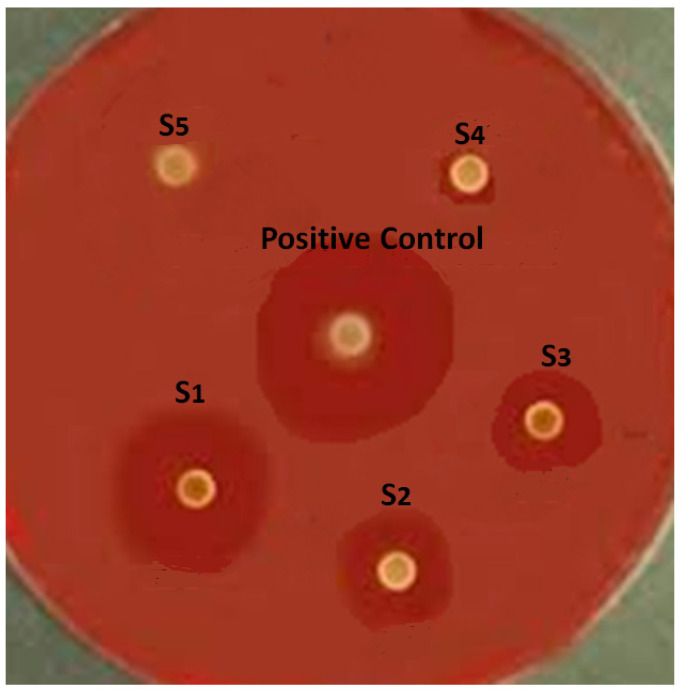
Mean growth inhibition zone of curcumin nanoparticle and control antibiotic in disk diffusion test.

The results of in vitro microbial tests showed that *P. gingivalis* was sensitive to the suspension of curcumin nanoparticles at concentrations of 50, 25, 12.5, and 6.25 µg/mL (Figure 2).

The results of the one-way ANOVA (between curcumin groups) showed that there is a significant difference between the curcumin groups for the mean growth inhibition zone, and curcumin nanocrystals with a concentration of 50 µg/mL showed the highest inhibition zone (*p* = 0.0003). In addition, the results of the one-way ANOVA (between all groups) showed that there is a significant difference between all groups for the mean growth inhibition zone, and metronidazole showed the highest inhibition zone (*p* = 0.0025).

### 3.4. Determination of MIC

**Figure 3:**
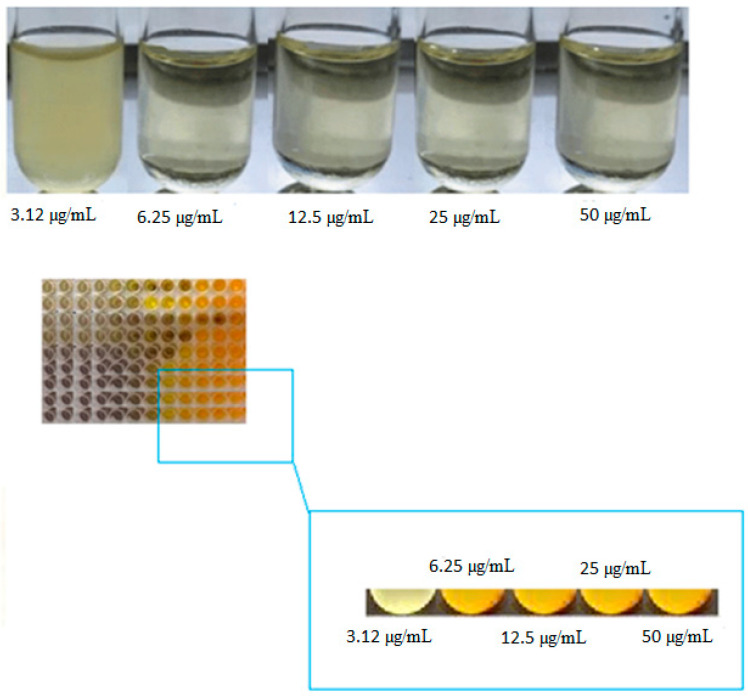
MIC results for curcumin nanoparticles against *P. gingivalis*.

Based on the MIC test, curcumin nanoparticles showed inhibitory effects against *P. gingivalis* at 6.25 µL/mL (Figure 3).

### 3.5. Determination of MBC

**Figure 4:**
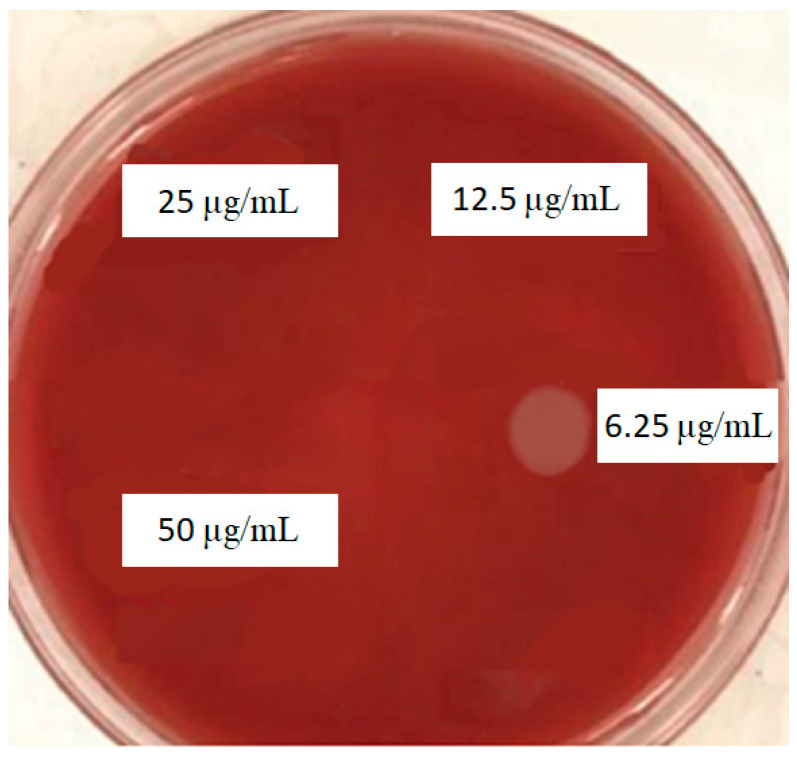
MBC results for curcumin nanoparticles against *P. gingivalis*.

Based on the MBC test, curcumin nanoparticles showed bactericidal effects against *P. gingivalis* at 12.5 µL/mL (Figure 4).

## 4. Discussion

Implant failure is a dramatic problem that leads to prosthesis failures as well [18]. It can occur due to various reasons including infection, problems with mechanical possessions of the implant [5], poor implant planning [5,6,7], wrong positioning, wrong prosthodontics plan, and too short biologic width [3].

One of the main worries for the clinical use of curcumin is its low bioavailability. Furthermore, information about its safety in higher doses is so little. Nanotechnology-based new plans are being discovered to improve curcumin’s bioavailability and decrease its toxicity [3,25]. In this study, curcumin nanoparticles showed an MIC of 6.25 µg/mL and an MBC of 12.5 µg/mL against *P. gingivalis*. Our microbial results in this study were different from previous reports using *P. gingivalis* (ATCC33277). Mandroli and Bhat showed that the MIC of curcumin against *P. gingivalis* (ATCC33277) was 125 μg/mL [26]. Shahzad et al. [27] also reported that the growth of *P. gingivalis* (ATCC33277) was inhibited by curcumin at a concentration of 7.8 μg/mL, whereas Izui et al. [28] found that *P. gingivalis* growth was inhibited at 20 μg/mL of curcumin. A recent study also reported that *P. gingivalis* (ATCC33277) was sensitive to a curcumin concentration of 100 μg/mL [29]. The difference between the results of the above-mentioned studies and our study can be explained by the fact that they used free curcumin against standard bacteria, whereas we used curcumin nanoparticles against clinically isolated bacteria.

In two other studies, the MIC and MBC values for curcumin were equal against clinically isolated *P. gingivalis*. According to the authors, it can be explained by the fact that curcumin showed antibacterial activity against *P. gingivalis* instead of bacteriostatic activity [19,30].

Some reports showed that curcumin inhibits bacteria by damaging the bacterial membrane [31]. Curcumin has also been revealed to inhibit bacterial cell proliferation by perturbation of the FtsZ assembly. Other reports also confirmed that curcumin can meaningfully inactivate bacteria by inducing the production of ROS, including singlet oxygen and hydroxyl radicals [32,33].

Depending on the particle size and kind of bacteria, nanoparticles apply their antibacterial effects on bacteria by numerous mechanisms. The nanoparticles used in this study had an average particle size of 95 nm. Reports have shown the main role of physicochemical properties (size, shape, and surface properties) and doses of nanoparticles in their antimicrobial actions [34]. Nanoparticles can disorder bacteria cell membrane functions by binding to the surface of cell membranes with a high affinity compared with larger nanoparticles. This function is more predominant in smaller nanoparticles owing to their larger surface area [35,36]. The interaction of bacteria’s membrane and nanomaterials originates in the local pores in the membrane. The entry of the nanoparticles into the bacteria also leads to intracellular damage of proteins (particularly protein-rich in sulfur) and DNA. Nanomaterials can also bind to the bacterial membrane and slowly enter the cytoplasm to disrupt bacterial functions. Some antibiotic-loaded nanomaterials can also blend their structure with the bacterial cell wall and insert their drug material into the cytoplasm [21,37].

## 5. Conclusions

The use of curcumin nanoparticles may control the bacterial infection around the implant. However, more animal and clinical studies using different concentrations of curcumin and different formulations are needed to improve its ability to inhibit early periodontal pathogens in cases of chronic periodontitis and to increase the stability of implants for a longer period. Unfortunately, to date, some studies on the benefits of curcumin have inconclusive results. In addition, little information is available to determine its safety in higher doses. Therefore, further investigation is needed from this point of view as well.

## 6. Suggestions

The culture method is the gold standard method for *P. gingivalis* bacteria and is always recommended as the first diagnostic step. However, using the combined molecular method can help for the easy identification of *P. gingivalis*.

## Data Availability

The raw/processed data can be shared by request from the corresponding author.
